# Subclinical Carotid Atherosclerosis Is Present from Early COPD Stages, Being in a Close Relationship with Systemic Inflammation

**DOI:** 10.3390/jcm15010180

**Published:** 2025-12-26

**Authors:** Ioana Ciortea, Emanuela Vastag, Corneluța Fira-Mladinescu, Alexandru Florian Crisan, Norbert Wellmann, Ana Adriana Trusculescu, Nicoleta Sorina Bertici, Daniel Traila, Cristian Oancea, Ovidiu Fira-Mladinescu

**Affiliations:** 1 Doctoral School, “Victor Babes” University of Medicine and Pharmacy, 2 Eftimie Murgu Square, RO-300041 Timisoara, Romania; ioana.ciortea@umft.ro (I.C.); norbert.wellmann@umft.ro (N.W.); 2 Center for Research and Innovation in Personalized Medicine of Respiratory Diseases (CRIPMRD), “Victor Babeș” University of Medicine and Pharmacy, 2 Eftimie Murgu Square, RO-300041 Timisoara, Romania; 3 Department of Biology and Life Sciences, “Vasile Goldis” University, 94 Revolutiei Boulevard, RO-310002 Arad, Romania; 4 Pulmonology University Clinic, Clinical Hospital of Infectious Diseases and Pneumophthisiology “Dr. Victor Babeș”, 13 Gheorghe Adam Street, RO-300310 Timisoara, Romania; 5 Center for Studies in Preventive Medicine, Hygiene Department, “Victor Babeș” University of Medicine and Pharmacy, 16 Victor Babeș Boulevard, RO-300226 Timisoara, Romania; 6 Research Center for the Assessment of Human Motion, Functionality and Disability (CEMFD), “Victor Babes” University of Medicine and Pharmacy, 2 Eftimie Murgu Square, RO-300041 Timisoara, Romania

**Keywords:** airflow obstruction, carotid intima–media thickness, atherosclerosis, inflammatory biomarkers

## Abstract

**Background:** Several cohort studies have demonstrated a link between subclinical carotid atherosclerosis and obstructive chronic airflow limitation. These conditions exhibit common risk factors associated with unhealthy lifestyles, as well as analogous pathophysiological mechanisms, including chronic low-degree systemic inflammation. **Purpose:** The aim of this study was to investigate the association between airflow obstruction and carotid intima–media thickness (c-IMT), together with the influence of inflammatory biomarkers on this relationship, in patients diagnosed with chronic obstructive pulmonary disease (COPD). **Methods and Patients:** This study is cross-sectional and includes 106 patients with stable COPD. All patients underwent evaluation through spirometry, carotid ultrasound, and assessment of inflammatory biomarkers, including C-reactive protein, fibrinogen, and erythrocyte sedimentation rate. The relationship between carotid subclinical atherosclerosis and the Global Initiative for Chronic Obstructive Lung Disease (GOLD) stage of COPD was assessed. Additionally, we compared patients with two positive biomarkers of inflammation with those who had no positive inflammatory biomarkers. **Results:** Significant statistical differences were observed in carotid intima–media thickness values associated with the severity of airflow obstruction, with measurements of 1.03 mm in COPD stage 1–2 GOLD, 1.07 mm in COPD GOLD 3, and 0.96 mm in GOLD 4 (*p* = 0.04). However, no direct correlation with forced expiratory volume in the first second (FEV1) was identified. The post hoc analysis revealed a notable increase in carotid wall thickness for the early stages of COPD. C-IMT demonstrated a significant association with inflammation parameters, muscle dysfunction, body composition, and lipid profile. The comparison of groups exhibiting two positive inflammatory biomarkers with those with no positive inflammatory markers revealed significant differences in age, c-IMT, exercise tolerance, and COPD symptoms. **Conclusions:** Subclinical carotid atherosclerosis is evident from the early stages of obstructive airflow limitation. Carotid intima–media thickness is significantly higher in patients with positive inflammatory biomarkers.

## 1. Introduction

Chronic obstructive pulmonary disease (COPD) is a heterogeneous pulmonary disorder marked by persistent respiratory symptoms resulting from airway and/or alveolar abnormalities that lead to persistent and frequently worsening airflow obstruction [1]. COPD constitutes a significant health issue, ranking as the third highest cause of mortality globally, following ischemic heart disease and stroke [2]. Currently, COPD is acknowledged as a systemic disease characterized by numerous extrapulmonary manifestations and comorbidities: 97.7% of patients present with at least one comorbidity, and 53% exhibit four or more [3,4]. The predominant comorbidities include cardiovascular diseases (CVDs), muscular dysfunction, metabolic disorders, osteoporosis, anxiety, and depression, all of which contribute to morbidity and mortality [5,6,7,8].

The association between CVDs and COPD has been extensively researched, revealing that CVDs are a primary cause of mortality in COPD, responsible for around 25 to 50% of deaths [9]. Moreover COPD is associated with elevated overall mortality and early death from CVDs, while impaired lung function correlates with higher cardiovascular risk [10,11]. COPD and CVDs share common risk factors, including smoking and pollution. However, while a significant link between compromised pulmonary function and early-stage vascular disease has been established in smokers, extensive cohort studies indicate that smoking alone insufficiently accounts for the additional cardiovascular risk in COPD patients [12,13]. Another potential explanation elucidating the greater prevalence of cardiovascular illness in individuals with COPD is the persistent presence of low-grade systemic inflammation, which is linked to elevated cardiovascular risk in the general population [14,15]. COPD exacerbations are associated with elevated systemic inflammatory biomarkers, including acute phase proteins, chemokines, and cytokines, and the exacerbating phenotype is recognized for its chronic systemic inflammation [16]. Chronic low-grade systemic inflammation occurs in a subset of patients with COPD [17]. The relationship between low-degree inflammation and the development of cardiovascular comorbidities still has many unexplored paths. COPD patients with persistent inflammation show a higher prevalence of cardiovascular comorbidities than those without a systemic inflammatory response, despite similar levels of pulmonary impairment [18]. Another very common invalidating systemic manifestation of COPD that is related to chronic systemic inflammation is muscle dysfunction; it reduces effort capacity, activity of daily life (DALY), patient autonomy, and finally quality of life [19]. Advanced and acute stages of COPD correlate with a heightened incidence of falls, balance deficits, systemic inflammation, and weakness in the lower extremities [20]. Muscle dysfunction is the result of a complex interaction between local factors, such as pulmonary hyperinflation, and systemic factors, such as aging, tobacco smoking, systemic inflammation, gas exchange abnormalities, anabolic insufficiency, and drugs [19,21].

The relationship between COPD and CVDs still remains largely unknown. A potential study area involves examining the associations between COPD and subclinical stages of CVDs, as well as identifying shared underlying pathophysiological variables, particularly those that are amenable to treatment or modification. Carotid intima–media thickness (c-IMT), ankle brachial pressure index, and cardio-ankle vascular index serve as surrogate indicators for detecting preclinical stages of CVDs [22,23,24,25]. Subclinical carotid atherosclerosis serves as a reliable predictor of future cardiovascular events, demonstrating a strong correlation with intracranial and coronary atherosclerosis within the general population [26,27]. On the other hand, elevated c-IMT correlates with increased total and cardiovascular mortality in COPD patients, indicating that carotid wall measurement could serve as a valuable marker for morbidity and mortality in this population [28,29]. Ultrasound has become a widely utilized first-line diagnostic tool for measurements of c-IMT and is recommended as part of cardiovascular assessment in patients with COPD [29,30].

Building on existing evidence regarding the presence of subclinical atherosclerosis in patients with COPD and the contributory role of persistent systemic inflammation, the present study was designed to investigate the association between the severity of airflow obstruction and c-IMT in patients with COPD. A secondary objective was to evaluate the modifying effect of systemic inflammatory biomarkers on this relationship.

## 2. Materials and Methods

### 2.1. Study Design and Participants

**Figure 1:**
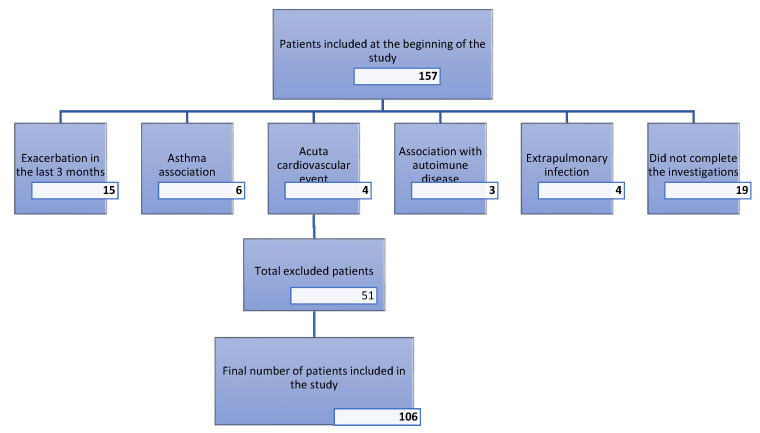
Flowchart for exclusion criteria.

This cross-sectional study included 106 patients with stable COPD (both men and women) who were under regular follow-up at our center. The median age of participants was 65 ± 9 years, with the majority having significant exposure to cigarette smoke exceeding 10 pack-years. The exclusion criteria included the following: exacerbation within the last three months, association with asthma, presence of any acute infection, systemic inflammatory disease, autoimmune disease, acute cardiovascular disease, and active neoplasia. Figure 1 illustrates the flow of participants through the study. A total of 157 patients were initially assessed for eligibility. Of these, 51 patients were excluded for the following reasons: 15 due to acute COPD exacerbations within the last three months, 19 who did not complete the investigations, 6 with an asthma association, 4 with acute cardiovascular events, 3 with autoimmune diseases, and 4 with extrapulmonary infections. Consequently, the final study population comprised 106 patients. Patients experiencing exacerbations were excluded regardless of severity, including mild episodes. All eligible patients gave written, informed consent prior to data registration. No invasive procedures were conducted in this study.

### 2.2. Patient Assessment

All patients received the subsequent assessment: registration of age and gender; anthropometric measurements, including height, weight, and body mass index (BMI) calculation; assessment of resting vital signs such as blood pressure, peripheral oxygen saturation, and heart rate; functional pulmonary evaluation through spirometry, alongside the determination of maximal respiratory pressures: maximum inspiratory pressure (MIP) and maximum expiratory pressure (MEP); imaging tests comprising posteroanterior chest radiography and carotid ultrasonography; blood sampling analyses for inflammatory biomarkers and lipid profile; evaluation of exercise performance via the six-minute walking test (6MWT); and assessment of body composition through dynamometry and bioelectrical impedance analysis (BIA). The assessment utilized a questionnaire approach, employing the modified Medical Research Council dyspnea scale (mMRC) to quantify dyspnea, the COPD assessment test (CAT) to evaluate the impact of COPD symptoms on quality of life, and the Hospital Anxiety and Depression Scale (HADS).

The diagnosis of COPD and the staging of airflow limitation severity were conducted in accordance with the GOLD (Global Initiative for Chronic Obstructive Lung Disease) strategy, utilizing forced spirometry to assess forced expiratory volume in the first second (FEV1), forced vital capacity (FVC), and the FEV1/FVC index. The stages are defined as follows: FEV1 > 80% corresponds to GOLD stage 1, FEV1 between 50% and 79% corresponds to GOLD stage 2, FEV1 between 30% and 49% corresponds to GOLD stage 3, and FEV1 < 30% corresponds to GOLD stage 4. All patients maintained a FEV1/FVC ratio of less than 0.7 following the administration of an inhaled short-acting bronchodilator (400 mcg of salbutamol). All measurements met the American Thoracic Society/European Respiratory Society (ATS/ERS) criteria for repeatability and reproducibility. MIP and MEP were evaluated using a shutter module integrated into a computer-based spirometer to assess the maximal efforts of the respiratory muscles.

High-resolution B-mode ultrasonography utilizing a linear transducer was performed to scan the bilateral carotid arteries. Three distinct longitudinal projections were analyzed: anterior oblique, posterior oblique, and lateral. c-IMT was assessed at the point of maximum thickness, as well as 1 cm upstream and 1 cm downstream from this site. The thickness for the three points for each carotid artery was determined, with the highest value of IMT being considered. Lesions with focal intima–media thickening exceeding 1.2 mm were classified as atherosclerotic plaques. All measurements were conducted using identical equipment and by the same physician, who remained unaware of the patients’ severity of COPD. The posteroanterior chest radiograph was included in the routine evaluation of each patient to rule out the presence of consolidation, such as pneumonia or cancer.

Blood samples were collected following a 12 h fast and analyzed for inflammatory biomarkers, specifically C-reactive protein (CRP), erythrocyte sedimentation rate (ESR), fibrinogen, and lipid profile components, including total cholesterol (TC), low-density lipoprotein (LDL) cholesterol, high-density lipoprotein (HDL) cholesterol, and triglycerides (TGs). The reference ranges for inflammatory biomarkers are as follows: CRP levels between 0 and 5 mg/L, ESR between 6 and 15 mm/h, and fibrinogen levels between 1.7 and 4.2 g/L. For the lipid profile, the normal values are as follows: total cholesterol < 200 mg/dL, LDL cholesterol < 100 mg/dL, HDL cholesterol > 40 mg/dL, and triglycerides < 150 mg/dL.

The 6MWT was conducted to assess the functional status of patients under the supervision of a physiotherapist, following the guidelines provided in the ATS statement for the 6MWT. A dynamometer was utilized for the hand grip test, which determines the maximum isometric strength of the hand and forearm muscles. A bioelectrical impedance analyzer was used to assess the body composition of the patients, specifically measuring body fat and muscle mass.

Systemic inflammation is not clearly defined in COPD, and its prevalence largely depends on the specific inflammatory marker or set of markers assessed [17]. Circulating inflammatory biomarkers, including CRP, interleukin-6 (IL-6), tumor necrosis factor-α (TNF-α), ESR, and fibrinogen, are frequently elevated in patients with stable COPD and have been consistently associated with greater disease severity, impaired lung function, and an increased burden of cardiovascular comorbidities [30,31,32]. Among these, serum fibrinogen, a well-established systemic marker linked to coronary heart disease, has been repeatedly reported to be elevated in COPD populations [32]. Furthermore, a systematic review and meta-analysis encompassing fourteen original studies demonstrated that systemic inflammation, as assessed by circulating biomarkers such as CRP and fibrinogen, is associated with lower FEV_1_ and FVC values [33]. In accordance with current recommendations for the definition of persistent systemic inflammation in stable COPD, which emphasize the concurrent positivity of at least two serum inflammatory biomarkers at two consecutive assessments, and taking into account the widespread availability of CRP, ESR, and fibrinogen in routine clinical practice, the present study compares patients exhibiting ≥2 positive inflammatory biomarkers among these three markers with those showing no evidence of systemic inflammation, defined by the absence of elevated inflammatory biomarkers [17].

### 2.3. Statistical Analysis

Data collection and analysis were performed using GraphPad Prism 9.3.0 software. Continuous variables with a Gaussian distribution are presented as means with standard deviations, while variables without a Gaussian distribution are presented as medians with interquartile ranges from the 25th to the 75th percentiles. A Receiver Operating Characteristic (ROC) curve analysis was conducted to determine the optimal threshold value of c-IMT for predicting the presence of atheromatous plaques in the patient cohort. One-way Analysis of Variance (ANOVA) and unpaired Student’s *t*-tests were employed to assess the significance of differences between groups for parametric variables, while the Mann–Whitney test was utilized for non-parametric variables. The chi-square test was used to compare frequencies. To assess the potential association between two variables, the Pearson coefficient of correlation (r) was calculated. For evaluating potential determinants of c-IMT, multiple linear regression analysis was performed. The selection of independent variables was based on their associations observed in the univariate analyses and other clinically relevant cardiovascular risk factors.

## 3. Results

**Figure 2:**
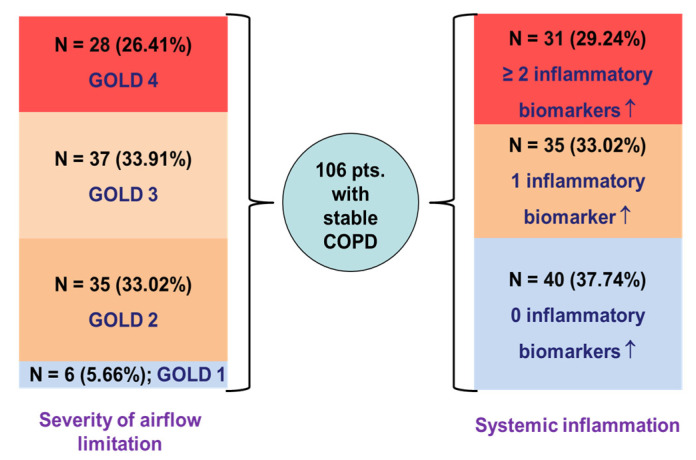
Distribution of COPD patients according to severity of airflow obstruction and systemic inflammation status; ↑—elevated values.

The distribution of COPD patients according to GOLD grades and the number of elevated inflammatory biomarkers is shown in Figure 2.

Table 1 illustrates the cohort characteristics for each measured parameter. As can be seen, COPD patients exhibit a median of three comorbidities, the most prevalent being cardiovascular disorders, with 78% of patients exhibiting at least one clinically evident cardiovascular condition.

**Figure 3:**
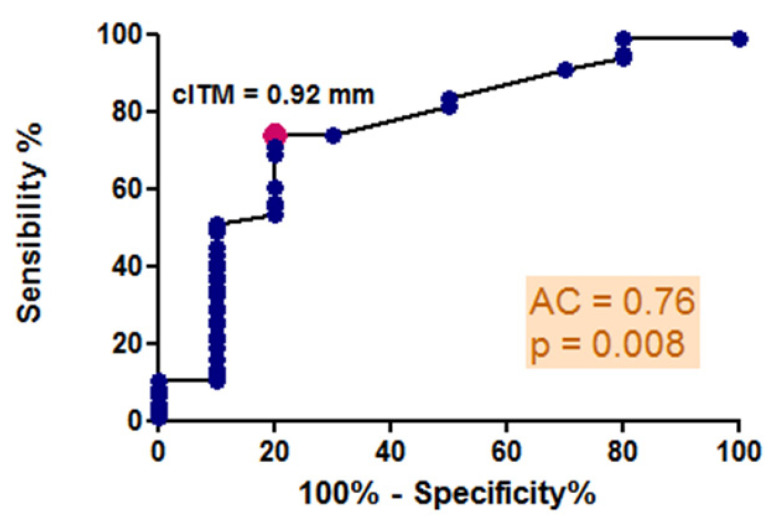
ROC curve analysis: predictive cutoff value for the presence of carotid atheromatous plaques: sensibility and specificity.

**Figure 4:**
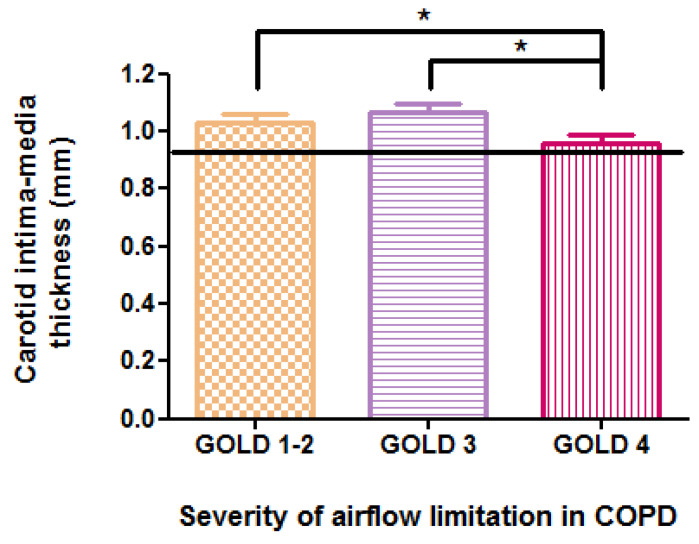
Relationship between carotid intima–media thickness and COPD severity according to GOLD staging; * *p* < 0.05.

The mean values of c-IMT, as measured by high-resolution B-mode ultrasonography, exceeded 0.92 mm across all stages of COPD. This value represents the optimal predictive cutoff for the presence of atheromatous plaques, as indicated by our ROC curve analysis, illustrated in Figure 3, which generated an area under the curve of 0.76, demonstrating good diagnostic accuracy for the test. Significant differences in c-IMT values were observed in relation to the severity of COPD, with measurements of 1.03 ± 0.14 mm in stage 1–2 GOLD, 1.07 ± 0.17 mm in stage 3 GOLD, and 0.95 ± 0.18 mm in stage 4 GOLD (*p* = 0.04) (Figure 4). However, no direct correlation was found with FEV1 (r = 0.10, *p* = 0.33), and post hoc analysis indicated a more significant carotid wall thickness in the early stages (*p* < 0.05). The results indicate that the greatest c-IMT value was recorded in the GOLD 3 stage of COPD, not in GOLD 4 stage, which may be considered counterintuitive. The potential reasons will be presented in the subsequent discussion.

Table 2 presents the relationship between the various measured variables and c-IMT, along with their statistical significance. As we expected, c-IMT demonstrated significant correlations with several traditional atherosclerosis risk factors, including age (r = 0.36, *p* < 0.001), BMI (r = 0.25, *p* = 0.01), body fat (%) (r = 0.34, *p* < 0.001), LDL cholesterol (r = 0.20, *p* = 0.04), and HDL cholesterol (r = −0.19, *p* < 0.05). A significant correlation was observed between c-IMT and the muscular dysfunction profile: MIP (r = −0.24, *p* = 0.02), MEP (r = −0.21, *p* = 0.04), hand grip force (r = −0.26, *p* = 0.02), 6MWT distance expressed in meters (r = −0.23, *p* = 0.02), and FEF_50%_/FVC, which is a marker of distal airflow obstruction.

**Figure 5:**
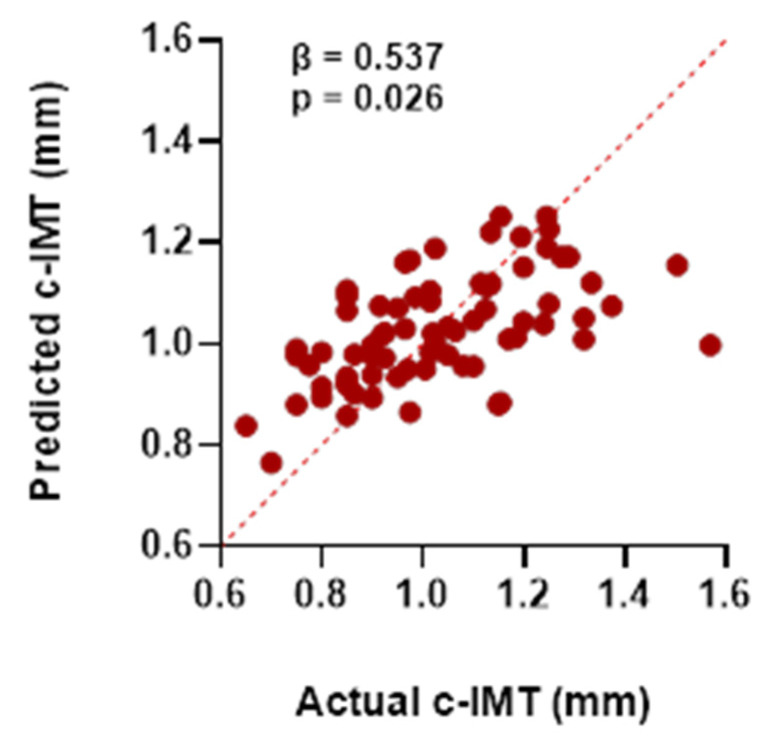
Scatter plot showing the relationship between observed and predicted carotid intima–media thickness (c-IMT) values obtained from the multiple linear regression model. The intercept coefficient (β_0_) and its corresponding *p*-value are displayed within the plot. The red dots represent individual data points. The red dashed line indicates a strong agreement between the actual and predicted values.

Figure 5 and Table 3 present the results of the multiple linear regression analysis assessing potential determinants of c-IMT. The selection of independent variables was based both on their associations observed in the univariate analyses and other clinically relevant cardiovascular risk factors. Figure 5 illustrates the relationship between observed and predicted c-IMT values, displaying the model fit together with the intercept coefficient (β_0_) and its *p*-value. Table 3 summarizes the regression coefficients (β), 95% confidence intervals, and *p*-values for all predictors included in the multivariable model.

In multivariate linear regression analysis, which included sex, age, smoking history (pack-years), body mass index, body fat percentage, FEF_50%_/FVC ratio, MIP, handgrip force, LDL cholesterol, and 6 min walk distance, age was the only independent predictor associated with c-IMT. It was positively and significantly associated with c-IMT values (β = 0.005, 95%, *p* = 0.04). It is worth mentioning that for the FEF_50%_/FVC ratio, the correlation observed in the univariate analysis lost its statistical significance in the multivariate analysis. Because there is no clear definition of inflammatory COPD phenotype, we compared the group with at least two positive inflammatory biomarkers among CRP, ERS, and fibrinogen with the group without any positive inflammatory biomarkers to see if there were any statistically significant differences. Significant statistical differences between the two groups were demonstrated regarding carotid atherosclerosis (*p* = 0.01), parameters of muscle dysfunction (6MWD (*p* < 0.01)), and patient age (*p* = 0.02). Table 4 shows the main differences between the groups.

## 4. Discussion

Our results showed that subclinical carotid atherosclerosis is present form early stages of obstructive airflow limitation. Carotid intima–media thickness is significantly higher in COPD patients showing two positive inflammatory biomarkers, and it is closely related to age, chronic hypoxia, and exercise capacity.

The mean c-IMT values were above the optimal predictive threshold for atheromatous plaque detection across all COPD stages. Although c-IMT varied significantly with COPD severity, it was not directly associated with FEV_1_ in univariate analysis, but only with FEF_50_%/FVC, a marker of small airway obstruction. This association lost significance in multivariate analysis, and intriguingly, the linear regression was positive, suggesting that patients with less severe airflow limitation exhibited greater carotid wall thickening. Large cohort studies evaluating the relationship between impaired pulmonary function and c-IMT have reported comparable findings. The Multi-Ethnic Study of Atherosclerosis (MESA) Lung Study and the Atherosclerosis Risk in Communities (ARIC) Study showed that the decrease in FEV1 is linked to an increase in c-IMT [34,35,36,37]. The Rotterdam Study indicated that COPD patients have a twofold increased risk of carotid wall thickening compared to the general population [38]. Another recent study concluded that there is an inverse association between c-IMT and lung function test score in COPD patients in comparison with control patients [39]. Our post hoc analysis indicated a significantly increased carotid wall thickness in the early stages of disease. Actual evidence suggests that endothelial dysfunction, which is one of the underlying mechanisms of atherosclerosis, occurs in early stages of COPD and worsens with pulmonary obstruction severity [40]. In addition, there is evidence that biomarkers of endothelial dysfunction are elevated in COPD patients and are negatively correlated with FEV1 [41,42]. All these results, including the results from our study, demonstrate the implications of obstructive airflow limitation in the pathogenesis of subclinical carotid atherosclerosis.

As mentioned earlier, a paradoxical result was obtained: the greatest value for c-IMT was observed in stage 3 of the disease, not in stage 4. In this study we included stable COPD patients, and it is known that the prevalence of CVDs is increased in patients with clinically stable COPD but also that CVDs are a prominent cause of death, particularly in patients with mild to moderate airflow obstruction [43]. Also, it was demonstrated that non-exacerbating patients with COPD died less of respiratory causes than exacerbators and more of malignancies and CVDs [44]. Moreover, a substantial cardiovascular burden and early mortality was demonstrated in patients with a preserved FEV1/FVC but impaired spirometry (decreased FEV1) before these patients developed COPD [45]. This means that survivor bias may contribute the following: patients with the poorest cardiovascular profiles, including the most advanced subclinical atherosclerosis, may be underrepresented in the GOLD 4 population due to earlier cardiovascular morbidity and mortality.

The physiopathology of the association between c-IMT and airflow limitation is still unclear: oxidative stress and chronic hypoxia may contribute, but the most obvious factor is thought to be chronic low-degree systemic inflammation [40,42]. For this reason, the second aim of the study was to compare the groups of COPD patients regarding the positivity of inflammatory biomarkers. In this study 29.24% of patients had two or more positive inflammatory biomarkers, and in this group c-IMT was significantly higher than in the group of patients negative for inflammatory biomarkers. The mean value of c-IMT in the inflammatory group was 1.15 ± 0.25 mm, while in the group without positive inflammatory biomarkers it was 1.02 ± 0.17 (*p* = 0.01). Several large population-based studies have reported that inflammatory biomarkers, such as CRP levels, were not associated with IMT but rather with plaque prevalence, whereas FEV_1_ showed no relationship with carotid plaque occurrence but was significantly correlated with IMT [46,47,48].

In our study, c-IMT demonstrated a strong positive association with traditional atherosclerotic risk factors—BMI, fat body weight (FBW), and LDL cholesterol—even though the mean values of these variables were comparable between the groups defined by inflammatory biomarker status. Previous studies also showed that carotid intima–media thickness was higher in people with a higher BMI [49]. Furthermore, large studies have demonstrated that elevated low-density lipoprotein is associated with higher maximum c-IMT levels in both women and men, even in the young adult population [50,51]. On the other hand, a study that analyzed the lipid composition of COPD patients showed that LDL levels were significantly elevated in COPD patients compared to the control group [52]. In this context, with respect to the comparison of the mean c-ITM between groups in the present study, the similarity in terms of BMI, FBW, and LDL cholesterol is important, reducing possible bias and underlining the implications of inflammatory biomarkers in carotid atherosclerosis pathogenesis.

Another important difference between the groups was the age of the patients. For the group with two positive inflammatory biomarkers, the median was 66 ± 8 years, while for the control group the value was 61 ± 9 years, this difference being statistically significant (*p* = 0.02). It is already know that aging is associated with low-degree systemic inflammation, the concept of inflammaging having been introduced in 2000 by Professor Francheschi to define a progressive increase in proinflammatory status related to the aging process [53,54]. This finding suggests that aging plays an important role in COPD patients regarding the level of systemic inflammation.

Beyond its association with systemic inflammation, aging itself appears to play a central role in vascular remodeling and carotid intima–media thickening. Data from the literature indicate that age is one of the strongest determinants of c-IMT, showing a strong linear association with increasing intima–media thickness independent of comorbidities [55]. This finding is consistent with our results, where age emerged as the only independent predictor of c-IMT in multivariate analysis, and is supported by large cohort studies reporting progressively higher c-IMT values with advancing age, suggesting the need for age-specific cutoff values [56]. Lung function also declines with aging in individuals without respiratory disease, while this decline is more pronounced in patients with COPD; in older adults with COPD, the annual reduction in FEV_1_ is substantially greater than in age-matched individuals without COPD [57]. Collectively, these data indicate that both c-IMT and FEV_1_ are strongly influenced by the aging process.

In individuals with COPD, airflow limitation results in persistent hypoxia of the vascular walls. The hypoxia of the arterial wall triggers the activation of a cascade of epithelial growth factors and cytokines, resulting in alterations such as enhanced vascular permeability and heightened platelet adhesion, and it also leads to the accumulation of hypoxia-inducible factor (HIF)-1α, which mediates many of the hypoxia-induced processes during plaque initiation and growth. These alterations result in augmented proliferation and division of intimal cells, thereby increasing the thickness of the intima–media of the vessels [58]. In our investigation, patients with two positive inflammatory biomarkers had both elevated c-IMT and reduced oxygenation levels, with the differences between groups being statistically significant, indicating an intricate relationship between chronic hypoxia and proinflammatory status. Furthermore, hypoxia induces endothelial cell responses that contribute to the pathogenesis of various diseases, including cardiovascular and lung diseases, but also chronic inflammation [59].

In the group of individuals presenting two positive inflammatory biomarkers, a significantly reduced distance was found during the 6MWT. Also, the median values for MIP, MEP, and hand grip strength were lower than in the group without positive inflammatory biomarkers, but the changes were not statistically significant. Concerning muscular dysfunction, analogous findings have been shown in other studies which indicate that MIP and SMIP (sustained maximum inspiratory pressure) diminish with increased inflammation, or that systemic inflammation appears to adversely affect muscle strength in patients with COPD [60,61]. These findings are significant, as randomized controlled trials have shown that individuals with COPD who adhere to an exercise training program exhibit not only better exercise tolerance but also improvements in systemic inflammation and endothelial dysfunction [62].

Taken together, the available evidence, including the findings of the present study, supports a strong association between COPD and the development of subclinical carotid atherosclerosis. These data underscore the contribution of COPD-related pathophysiological processes—particularly systemic inflammation and endothelial dysfunction—to early vascular remodeling. However, the independent contribution of airflow limitation to this relationship remains incompletely understood and warrants further investigation in well-designed longitudinal studies to disentangle the relative roles of pulmonary function impairment, systemic inflammation, and cardiovascular risk burden.

A possible limitation of the study is due to the fact that significantly more males than females were included in the study. This may have been due to the differences between genders in Romania regarding tobacco use. According to World Health Organization statistics for Romania, in 2000 the estimated tobacco use prevalence for both genders was 35.2% for males 47.3% and 23.9% for woman [63]. Another possible bias of this study is the relatively small number of patients classified as GOLD 1 stage. This imbalance is largely attributable to the well-recognized underdiagnosis of COPD in its earliest stage [64]. Many individuals in stage 1 received care in outpatient settings. Consequently, a considerable proportion of early-stage patients were lost to follow-up, reducing the representation of this subgroup in the analysis. Moreover, we used as inflammatory biomarkers CRP, ESR, and fibrinogen, excluding other valuable blood biomarkers, such as IL-6, IL-8, and TNF-α. This is due to the wide availability of the studied biomarkers in current clinical practice. Another possible limitation of this study is that we restricted by design the comparative analysis to patients with clearly defined inflammatory phenotypes, thereby excluding patients with a single positive biomarker. Given the broad spectrum of cardiovascular comorbidities associated with COPD—including arterial hypertension, coronary artery disease, myocardial infarction, atrial fibrillation, other arrhythmias, peripheral vascular disease, and heart failure—comparative analyses of individual cardiovascular disease subgroups were not performed [65]. Such stratification would have resulted in sample sizes too small to allow for robust statistical analysis.

## 5. Conclusions

This study demonstrated the presence of subclinical carotid atherosclerosis in early stages of COPD potentially resulting from low-grade systemic inflammation in stable patients.

More research is needed to fully understand the underlying mechanism of subclinical atherosclerosis in COPD patients and the contribution and effects of low-degree systemic inflammation.

## Figures and Tables

**Table 1:** Cohort characteristics.

Characteristic	Value
Male/female	95/11
Age (years)	65 ± 9
Smoking (pack-years)	42 ± 29
Comorbidities (*n*)	3 (2–4)
c-IMT (mm)	1.03 ± 0.18
BMI (kg/m^2^)	25.86 ± 6.03
Body fat (%)	24 ± 10
FBW (kg)	18.91 ± 11.30
LBW (kg)	52.26 ± 10.08
FVC (% pred.)	64 ± 19
FEV1 (% pred.)	46 ± 20
FEV1/FVC	0.53 ± 0.13
FEF_50%_/FVC	0.30 ± 0.18
MIP (% pred.)	55 ± 23
MEP (% pred.)	56 ± 23
SpO2 (%)	96 (94–98)
Hand grip force (kgf)	5.08 ± 1.5
CAT	19 (12–24)
mMRC scale	2 (1–3)
HADS anxiety	7 (5–11)
HADS depression	8 (5–12)
LDL cholesterol (mg/dL)	142 ± 54
HDL cholesterol (mg/dL)	55 ± 17
Triglycerides (mg/dL)	122 ± 66
ESR (mm/h)	10 (5–30)
Fibrinogen (g/L)	2.86 (2.40–3.29)
CRP (mg/L)	3.88 (2.10–10.14)
6MWD (m)	363 ± 155
6MWD (% pred.)	82 ± 41

Notes: Data are presented as means ± standard deviations (SDs) for normally distributed continuous variables and as medians with interquartile ranges (25th–75th percentiles) for non-normally distributed variables. Abbreviations: 6MWD = 6-min walking distance; BMI = body mass index; CAT = COPD assessment test; c-IMT = carotid intima–media thickness; CRP = C-reactive protein; ESR = erythrocyte sedimentation rate; FBW = fat body weight; FEF_50%_ = forced expiratory flow at 50% of FVC, FEV1 = forced expiratory volume in the first second; FVC = forced vital capacity; HADS = Hospital Anxiety and Depression Scale; HDL = high-density lipoprotein; LBW = lean body weight; LDL = low-density lipoprotein; MEP = maximum expiratory pressure; MIP = maximum inspiratory pressure; mMRC = medical Modified Research Council, SpO2 = peripheral oxygen saturation.

**Table 2:** Association of multiple analyzed variables with c-IMT.

Parameters	R	*p*
Age (years)	0.360	0.0002 **
Smoking (pack-years)	0.141	0.160
BMI (kg/m^2^)	0.247	0.0164 *
Body fat (%)	0.343	0.001 **
FBW (kg)	0.342	0.001 **
LBW (kg)	0.108	0.302
FVC (% pred.)	−0.007	0.947
FEV1 (% pred.)	0.098	0.330
FEV1/FVC	0.177	0.077
FEF_50%_/FVC	0.262	0.009 *
MIP (% pred.)	−0.235	0.019 *
MEP (% pred.)	−0.211	0.035 *
SpO2 (%)	−0.069	0.504
Hand grip force (kgf)	−0.258	0.016 *
CAT	0.086	0.397
mMRC scale	0.118	0.235
HADS anxiety	0.191	0.063
HADS depression	0.074	0.475
LDL cholesterol (mg/dL)	0.202	0.039 *
HDL cholesterol (mg/dL)	−0.194	0.048 *
Triglycerides (mg/dL%)	0.133	0.179
ESR (mm/h)	0.094	0.342
Fibrinogen (g/dL)	0.142	0.154
CRP (mg/L)	0.063	0.536
6MWD (m)	−0.230	0.024 *
6MWD (% pred.)	−0.156	0.129

Notes: * *p* < 0.05 is statistically significant; ** *p* < 0.001 is highly statistically significant. Abbreviations: 6MWD = 6-min walking distance; BMI = body mass index; CAT = COPD assessment test; CRP = C-reactive protein; ESR = erythrocyte sedimentation rate; FBW = fat body weight; FEF_50%_ = forced expiratory flow at 50% of FVC; FEV1 = forced expiratory volume in the first second; FVC = forced vital capacity; HADS = Hospital Anxiety and Depression Scale; HDL = high-density lipoprotein; LBW = lean body weight; LDL = low-density lipoprotein; MEP = maximum expiratory pressure; MIP = maximum inspiratory pressure; mMRC = modified Medical Research Council.

**Table 3:** Multiple linear regression analysis of potential determinants of carotid intima–media thickness.

Variable	β (Coefficient)	95% Confidence Interval	*p*-Value
Sex (M/F)	−0.1264	−0.2762 to 0.0233	0.097
Age (years)	0.005002	0.00020 to 0.00981	0.042 *
Smoking (pack-years)	0.00007048	−0.001345 to 0.001486	0.921
BMI (kg/m^2^)	0.004940	−0.00620 to 0.01608	0.379
Body fat (%)	0.001241	−0.00577 to 0.00825	0.725
FEF_50%_/FVC	0.1808	−0.0651 to 0.4268	0.147
MIP (% pred.)	−0.0001975	−0.00223 to 0.00184	0.847
Hand grip force (kgf)	−0.01320	−0.04606 to 0.01966	0.425
LDL cholesterol (mg/dL)	0.0006386	−0.000291 to 0.001568	0.175
6MWD distance (m)	−0.0001185	−0.000422 to 0.000185	0.439

Abbreviations: 6MWD = 6-min walking distance, BMI = body mass index, FEF_50%_ = forced expiratory flow at 50% of FVC, MIP = maximum inspiratory pressure, LDL = low-density lipoprotein. * = statistical significance.

**Table 4:** Differences between groups regarding inflammatory biomarkers.

Parameters	Negative Inflammatory Biomarkers	Two Positive Inflammatory Biomarker	*p*-Value
Male/female (n)	36/4	29/2	0.594
Age (years)	61 ± 9	66 ± 8	0.015 *
Smoking (pack-years)	41 ± 25	48 ± 31	0.299
c-IMT (mm)	1.02 ± 0.17	1.15 ± 0.25	0.012 *
BMI (kg/m^2^)	24.67 ± 5.88	25.13 ± 5.73	0.746
Body fat (%)	21 ± 11	25 ± 11	0.231
FBW (kg)	16.92 ± 11.25	19.33 ± 12.03	0.418
LBW (kg)	53.55 ± 10.64	50.94 ± 8.23	0.294
FEV1 (% pred.)	44 ± 21	39 ± 15	0.290
FEV1/FVC	0.49 ± 0.12	0.54 ± 0.13	0.194
FEF_50%_/FVC	0.27 ± 0.18	0.30 ± 0.18	0.610
MIP (% pred.)	59 ± 24	56 ± 22	0.525
MEP (% pred.)	56 ± 19	54 ± 20	0.591
SpO2 (%)	96 (94–98)	94 (91–94)	0.034 *
Hand grip force (kgf)	5.41 ± 1.66	5.06 ± 1.30	0.396
CAT	17 (10–22)	22 (12– 25)	0.110
mMRC scale	2 (1–3)	2 (1–4)	0.113
HADS anxiety	7 (3–10)	8 (5–12)	0.141
HADS depression	7 (4–13)	9 (6–12)	0.139
LDL cholesterol (mg/dL)	135.0 ± 47.26	140.9 ± 66.90	0.665
HDL cholesterol (mg/dL)	57.56 ± 16.49	57.61 ± 21.74	0.992
TGs (mg/dL)	116.1 ± 63.25	111.7 ± 52.20)	0.758
6MWD (m)	416.9 ± 121.0	309.3 ± 153.6	0.002 *
6MWD (% pred.)	85 ± 25	65 ± 32	0.005 *

Notes: Data are presented as means ± standard deviations (SDs) for continuous variables with a Gaussian distribution and medians with interquartile ranges for variables with a non-Gaussian distribution. Abbreviations: 6MWD = 6-min walking distance; BMI = body mass index; CAT = COPD assessment test; c-IMT = carotid intima–media thickness; FBW = fat body weight; FEV1 = forced expiratory volume in the first second; FVC = forced vital capacity; HADS = Hospital Anxiety and Depression Scale; HDL = high-density lipoprotein; LBW = lean body weight; LDL = low-density lipoprotein, MEP = maximum expiratory pressure; MIP = maximal inspiratory pressure; mMRC = modified Medical Research Council; TGs = triglycerides; * = statistical significance.

## Data Availability

The data presented in this study are available on request from the corresponding author.

## Abbreviations

The following abbreviations are used in this manuscript:

6MWT	6-min Walking Test
6MWD	6-min Walking Distance
ARIC	Atherosclerosis Risk in Communities
ATS	American Thoracic Society
BIA	Bioelectrical Impedance Analysis
BMI	Body Mass Index
CAT	COPD Assessment Test
c-IMT	Carotid Intima–Media Thickness
COPD	Chronic Obstructive Pulmonary Disease
CRP	C-Reactive Protein
CVDs	Cardiovascular Diseases
DALY	Activity of Daily Life
ERS	European Respiratory Society
ESR	Erythrocyte Sedimentation Rate
F	Female
FEF_50%_	Forced Expiratory Flow at 50% of Forced Vital Capacity
FEV1	Forced Expiratory Volume in the First Second
FVC	Forced Vital capacity
FWB	Fatty Body Weight
GOLD	Global Initiative of Chronic Obstructive Pulmonary Disease
HADS	Hospital Anxiety and Depression Scale
HDL	High-Density Lipoprotein
HIF	Hypoxia-Inducible Factor
IL-6	Interleukin 6
IL-8	Interleukin 8
IMT	Intima–Media Thickness
LBW	Lean Body Weight
LDL	Low-Density Lipoprotein
M	Male
MESA	Multi-Ethnic Study of Atherosclerosis
MEP	Maximum Expiratory Pressure
MIP	Maximum Inspiratory Pressure
mMRC	Modified Medical Research Council
ROC	Receiver Operating Characteristic
SpO2	Peripheral Oxygen Saturation
TC	Total Cholesterol
TGs	Triglycerides
TNF-α	Tumor Necrosis Factor α
